# Timing matters: dental development and outcomes on secondary alveolar bone grafting in cleft lip and palate patients

**DOI:** 10.1007/s00784-025-06594-w

**Published:** 2025-10-30

**Authors:** Theodosia Bartzela, Isabel Hoffmann, Jennifer Kluge, Fabian Jäger, Michael Schmechel, Charlotte Opitz

**Affiliations:** 1 Department of Orthodontics, University Hospital Carl Gustav Carus, Technische Universität Dresden, Fetscherstr. 74, Dresden, Saxony 01307 Germany; 2 Private Orthodontic Practice, Berlin, Germany; 3 Private Oral and Maxillofacial Surgery Practice, Berlin, Germany

**Keywords:** Secondary alveolar bone grafting, Cleft lip and palate, Orthodontic space closure, Canine eruption

## Abstract

**Objective:**

This is the first study to systematically evaluate the timing of secondary alveolar bone grafting (SABG) based on canine root mineralization stages (R-value), assessing its impact on bone graft preservation, canine eruption, and space closure in patients with cleft lip and palate (CLP).

**Materials and methods:**

This retrospective single-center cohort study included 104 patients with unilateral or bilateral CLP (127 cleft sites). Orthopantomograms and clinical records were evaluated at three stages: pre-SABG, six months post-SABG, and post-orthodontic treatment. The R-value was classified as *R* = 0,25 − 1,0, corresponding to 25–100% root development, respectively. Assessed parameters included the canine mineralization stage, axis-angulation and vitality, limbus height, probing depths, and space closure strategy. Group comparisons were performed using t-tests.

**Results:**

Early SABG (*R* ≤ 0,5) yielded significantly higher orthodontic space closure rates (71% versus 25% with late SABG, *p* < 0,05) with better graft preservation, but was associated with increased canine impaction (18,6% in lateral incisor agenesis cases). The mean axis-angle of cleft-side canines differed significantly between orthodontic and prosthodontic space closure (81,3° vs. 91,0°, t = 5,702). Limbus alveolaris height was reduced when SABG occurred after root completion (*R* = 1,0, t = 4,234). Periodontal probing depths remained < 3 mm, and canine vitality was preserved in all groups.

**Conclusions:**

Early SABG, timed according to canine mineralization, supports alveolar bone preservation and space closure without compromising periodontal health.

**Clinical relevance:**

Tailoring SABG timing based on dental development can optimize orthodontic and prosthodontic outcomes in patients with CLP.

## Introduction

Alveolar bone grafting (ABG) is a critical surgical procedure introduced in 1952 [1] for patients with cleft lip and palate. It primarily aims to reconstruct the alveolar cleft, maintaining arch continuity and stability. ABG supports tooth eruption [2], prevents alveolar segment collapse, and facilitates orthodontic tooth movement. Additionally, ABG improves facial symmetry [3], enhances aesthetics, restores function, and provides a foundation for future prosthodontic rehabilitation [4, 5]. The timing of ABG plays a critical role in treatment outcomes. Primary ABG is typically performed until 2 years of age as part of comprehensive cleft care, promoting optimal bone regeneration and tooth support [6]. Secondary alveolar bone grafting (SABG) is generally recommended between 9 and 11 years of age, ideally before the eruption of the permanent canines, and represents the current standard [7, 8]. Some authors advocate for optimal timing, either before or during the eruption of the lateral incisor, to support periodontal health and arch symmetry [6, 9]. However, long-term outcomes of early versus late SABG remain controversial. Early SABG, performed between 4 and 7,6 years, has largely been abandoned due to concerns associated with compromised maxillary growth [6, 7]. Nonetheless, recent studies advocate that SABG performed around six years of age does not hinder midfacial growth [9, 10]. While chronological age alone is insufficient, individualized approaches, such as assessing lateral incisor or canine root mineralization (R-value) or the residual bone thickness over the adjacent crowns, offer a more tailored strategy [6]. Beyond its impact on growth, SABG timing also influences dental eruption patterns, particularly regarding the risk of canine impaction. The initial position and angulation of the canine are critical factors, as a greater inclination and a higher initial position increased the risk of canine impaction, primarily when bone grafting was performed after significant root maturation [11]. The challenge in clinical decision-making lies in balancing the benefits of early intervention with potential effects on skeletal growth and dental development. Improper timing may lead to complications, including orthodontic instability or insufficient bone formation for future dental implants [4]. Notably, lateral incisor agenesis emerged as a critical factor, with canine impaction being more prevalent in cases with lateral incisor agenesis. This suggests that the lateral incisor plays a guiding role in directing the canine along its eruption path [12].

Conventional two-dimensional (2D) dental radiographs are widely used to evaluate outcomes of secondary alveolar bone grafting. Nevertheless, they often systematically overestimate bone volume in the grafted alveolar clefts due to anatomical superimpositions and are unable to capture bucco-palatal bone dimensions [13–15]. Although cone-beam computed tomography (CBCT) enables three-dimensional (3D) volumetric analysis [16], many long-term data sets rely on conventional 2D imaging due to existing patient documentation and accessibility limitations.

This study evaluates the timing of SABG based on R-values and its impact on alveolar bone preservation, eruption patterns, periodontal health, and graft stability. Specific parameters assessed include alveolar bone height, periodontal probing depths, and canine inclination adjacent to the clefts. By comparing treatment outcomes across different developmental stages and space closure strategies, this study identifies areas for improvement in interdisciplinary care protocols and decision-making in orthodontic and prosthodontic rehabilitation of cleft sites. However, no prior research systematically evaluated SABG timing using R-values in relation to both bone preservation and canine eruption patterns.

Therefore, this study aims to address the gaps in the current understanding of optimal SABG timing in patients with CLP by investigating its influence on periodontal and dental outcomes, as well as structural graft stability, in orthodontic and prosthodontic reconstruction alternatives for the cleft region.

## Participants and methods

### Participants

This study retrospectively collected data from patients with unilateral or bilateral CLP (UCLP or BCLP) treated with SABG. The patients were born between 1976 and 1985, and their evaluation was performed between the ages of 8 and 14. All cleft-related interventions were conducted at the same center at Charité - Universitätsmedizin Berlin. A total of 104 patients with 127 cleft regions were included in the study. The mean patient’s age was 9,9 ± 1,6 years at the time of surgery. Eighty-two cleft regions showed agenesis of the lateral incisor.

The inclusion criteria of this study were as follows:

- Patients with UCLP or BCLP with or without agenesis of the lateral incisor.
- Treated from birth at Charité - Universitätsmedizin Berlin.
- Successful SABG was performed between 1984 and 1999 using autologous bone.
- Caucasian ethnic background.
- No associated congenital malformations, syndromes, or intellectual disabilities.
- No multiple tooth agenesis.
- Age at SABG ranged from 8 to 14 years.

### Treatment protocol

The treatment of patients with CLP at the Charité - Universitätsmedizin Berlin, Campus Virchow Klinikum, followed a standardized treatment protocol, as summarized in Table 1. If required, orthodontic expansion was performed before bone grafting. At Charité, autologous bone grafting is typically employed for SABG, with bone commonly harvested from the iliac crest and transplanted into the alveolar cleft. This study collected data before, after SABG, and after orthodontic treatment.

**Table 1:** Standardized surgical and orthodontic treatment protocol for patients with CLP treatment at the Charité - Universitätsmedizin Berlin, Campus Virchow

age	surgical treatment protocol	orthodontic treatment protocol
4–6 months 2–4 years 4–6 years 6–11 years 11–16 years	lip closure soft and hard palate closure secondary bone grafting (iliac crest bone)	infant orthopedics correction of crossbite and/or collapsed arch segments and/or class III malocclusion treatment tooth eruption guidance (Hotz protocol), correction of rotated upper central incisors, and crossbite (removable and/or functional appliances) multibracket appliance

### Data acquisition

Data from patients with CLP who underwent SABG were retrospectively analyzed. Orthopantomograms (OPTG) taken before and after SABG, as well as following orthodontic treatment, were evaluated for the following parameters:

- Canine mineralization stage.
- Inclination of the canine’s longitudinal axis.
- Canine impaction incidence.
- Canine eruption through the bone graft in both sagittal and transverse dimensions.
- Bone graft and limbus alveolaris height of teeth adjacent to the cleft.

Clinical assessment included:

- Periodontal probing depth using a 6-point pocket chart.
- Canine vitality test.

### Methods

The study adhered to the STROBE guidelines for observational studies. This retrospective cohort study was conducted in accordance with the ethical standards of the institutional and national research committee and with the 1964 Helsinki Declaration and its later amendments or comparable ethical standards. The institutional ethics committee of Charité - Universitätsmedizin Berlin approved the study (EA2/240/17).

The surgical intervention was considered successful if a continuous bone graft was radiographically discernible six months after secondary osteoplasty.

#### Radiographic analysis

OPTGs were taken at the following time points:

- T0: preoperative assessment (mean age: 10,0 ± 1,8 years).
- T1: six months post-secondary osteoplasty (mean age: 12,5 ± 1,7 years).
- T2: after completion of orthodontic treatment (mean age: 16,6 ± 1,6 years).

**Fig. 1:**
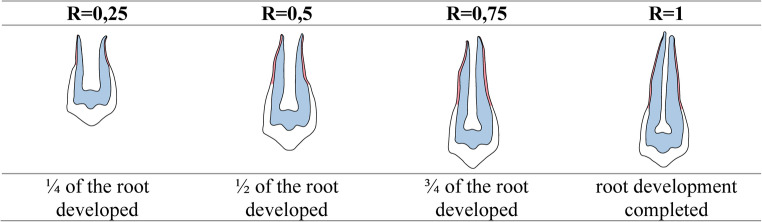
Example demonstrating canine root mineralization stages. R-values = root-to-crown ratio (dimensionless)

To minimize selection bias, only patients with complete and high-quality radiographic records were included in the analysis. Our retrospective study of all the eligible patients with cleft provided us a total of 127 OPTGs at T0 and T1. Moreover, 102 OPTGs or periapical radiograph regions from cleft areas were available at T2. The canine mineralization stage was determined using the method of Moorrees et al. [17] as modified by Schopf [18]. An adapted scheme is presented in Fig. 1. The limbus alveolaris height and bone graft were assessed using a modified version of the method described by Long et al. [19]. The axis angle of cleft-sided canines was measured following the method described by Dausch-Neumann [20].

OPTG provides broad availability, low radiation exposure, and sufficient resolution for routine clinical assessment. However, this method is inherently limited by its two-dimensional nature, which prevents assessment of bucco-palatal bone dimensions and volumetric resorption. Consequently, subtle 3D changes may not have been fully captured in this study.

#### Measurement procedures

**Fig. 2:**
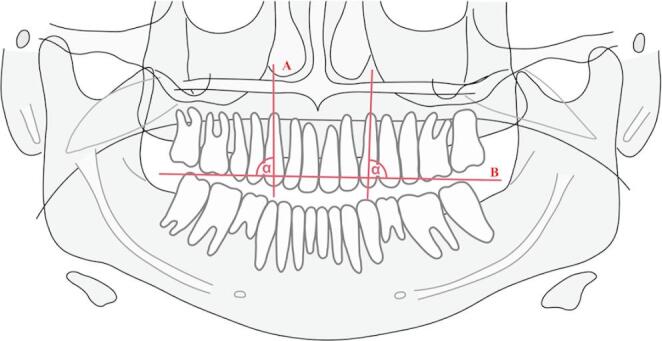
Representative OPTG demonstrating an axis-angle measurement between the canine axis and the occlusal plane. Method by Dausch-Neumann (1970) [20]. **A**: longitudinal canine axis, **B**: occlusal plane by Dausch-Neumann (1970) [20], connecting the distobuccal cusps of the first molars, α: canine axis-angle (in degrees °)

To ensure standardized and reproducible data, the following measurements were applied:

- Insertion height is defined as the distance from the cementoenamel junction to the cervical bone margin.
- Bone graft height: measured as the distance to the most apical point of the bone graft, perpendicular to the cementoenamel junction of the central incisor and the canine on the cleft side.
- Relative height of the limbus alveolaris and the bone graft: expressed as a dimensionless ratio based on one-tenth of the canine crown length.
- Canine axis-angle: defined as the angle enclosed between the occlusal plane (connecting the distobuccal cusps of the first molars) and the longitudinal axis of the canine (in the coronal-apical direction) [Fig. 2].

#### Orthodontic and clinical evaluation

- 3D plaster models (T2: 16,2 ± 1,7 years) of 64 cleft regions were used to measure the extent of orthodontic tooth movement during space closure via reflex microscopy.
- Clinical assessment of adjacent teeth at T3 (mean age: 17,7 ± 1,9 years) using the Parodontometer PCP 10 (Hu-Friedy©).
- Periodontal probing depth using a 6-point method on the teeth adjacent to the cleft was assessed at a clinical follow-up on 58 cleft regions, with registration performed per tooth.
- Canine vitality was assessed using carbon dioxide snow testing.

### Statistical analysis

All data were recorded and analyzed using SPSS (Version 6.0; Chicago, III). The following variables were evaluated:

- The axis-angle of the teeth adjacent to the cleft (in degrees).
- R-value was determined as a root-to-crown length ratio (dimensionless).
- Graft and limbus height of the alveolar ridge relative to the crown length (dimensionless).
- Model measurements for the distances (in millimeters).

The normal distribution was tested using the Kolmogorov-Smirnov test. Variance homogeneity was assessed with Levene’s test. Group comparisons between subgroups were conducted using Student’s t-test, applying the formula of Spiegel and Stephens [21]. We acknowledge that for small sample sizes (*n* < 30), non-parametric alternatives such as the Mann-Whitney U test may have been more appropriate; however, due to the retrospective design and for consistency of reporting, t-test results are presented throughout. A significance level of *p* < 0,05 was applied.

## Results

**Fig. 3:**
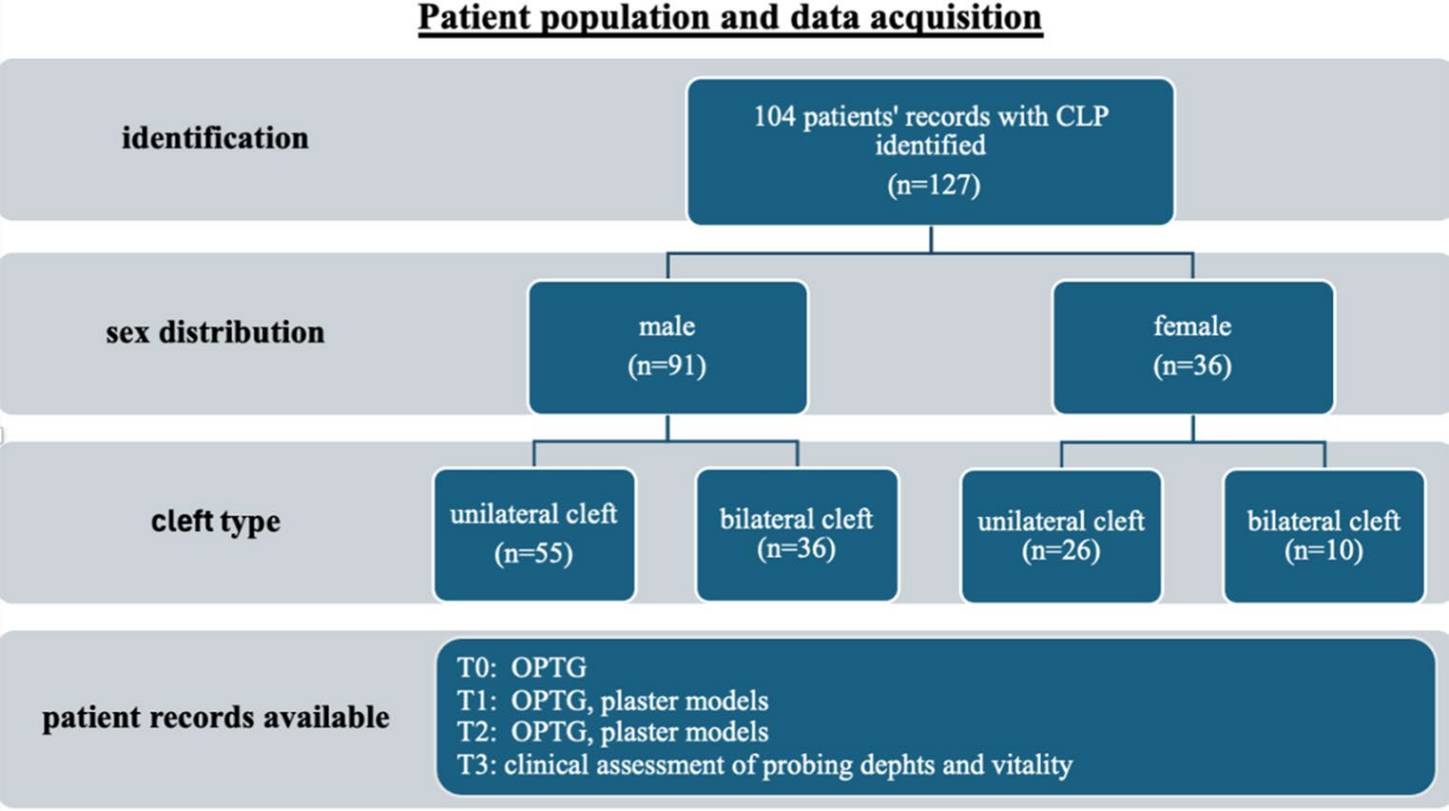
Patient distribution according to cleft type and sex, and records available at the registration time points T0: preoperative assessment, T1: postoperative assessment, T2/T3: after orthodontic treatment, n: number of cleft regions and patient distribution accordingly

In total, 104 patients with 127 cleft sites and a mean age of 9,9 ± 1,6 years at the time of surgery were included in the study. Eighty-two cleft sites showed agenesis of the lateral incisor. To facilitate statistical evaluation and data interpretation, the patient population was stratified based on the presence or absence of the lateral incisors and the type of space closure (orthodontic vs. prosthodontic). All patients were successfully treated with secondary osteoplasty using autologous bone grafting. The four stages of the R-value (R 0,25 − 1,0) were used to evaluate the optimal timing of SABG. Early and late SABG were used when bone grafting was performed at *R* ≤ 0,5 and *R* ≥ 0,5, respectively. Patient distribution according to cleft type and sex is presented in Fig. 3.

Of the 127 cleft sites, 81 (63,8%) were associated with unilateral cleft patients, while 46 (36,2%) involved bilateral clefts. The cohort included 91 males (71,6%) and 36 females (28,4%). The mineralization stage of canines adjacent to the cleft was age-dependent. The dental development of females was 0,2 − 0,5 years earlier than that of males. The sex distribution difference was statistically not significant.

### Space closure and timing of SABG

**Fig. 4:**
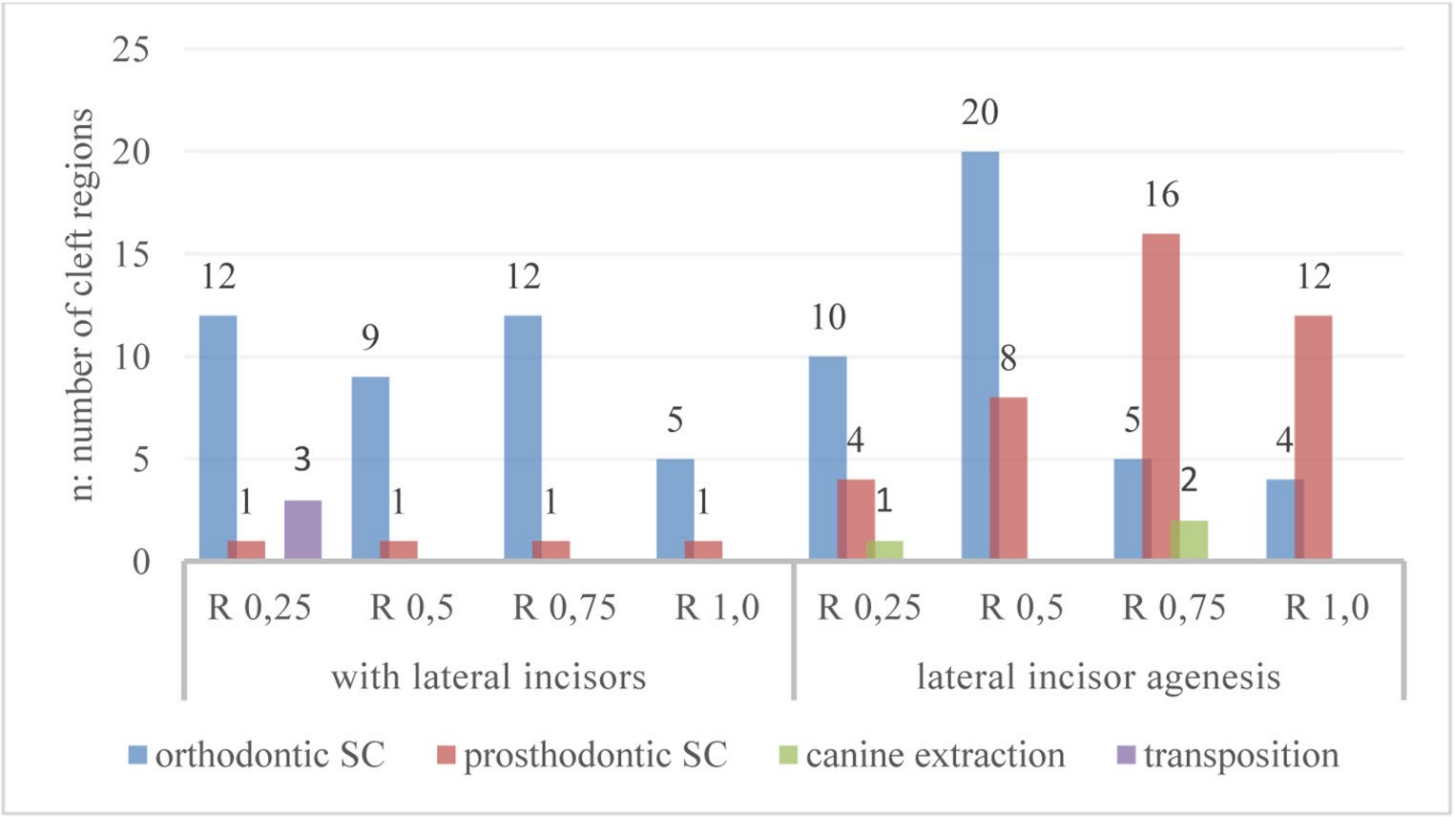
Distribution of orthodontic and prosthodontic space closure type (SC) according to R-value at the time of SABG, stratified by the presence or agenesis (with lateral incisors: n=45; lateral incisor agenesis: n=82). n = number of cleft regions; p < 0,05

Among the 127 cleft sites, 77 (60,6%) underwent orthodontic space closure, while 44 (34,6%) received prosthodontic rehabilitation. In three cleft regions, severe canine impaction and lateral incisor agenesis required surgical removal of the canine. Additionally, three cleft sites exhibited lateral incisor transposition with the canine, which occurred exclusively in early SABG cases (*R*≤0,5) [Fig. 4].

The success rate of orthodontic space closure in lateral agenesis patients was highest when SABG was performed at *R*≤0,5, with 66,6% at the mineralization stage *R* = 0,25 and 71,4% at stage *R* = 0,5. However, when SABG was performed later, during stages *R* = 0,75 and *R* = 1,0, the success rate for orthodontic space closure declined to only 21,8% and 25%, respectively.

However, the 82 cleft sites were nearly equally distributed between the two types of space closure: 43 (52,4%) received prosthodontic and 39 (47,6%) orthodontic space closure. The distribution of space closure type was equally balanced within these subgroups.

### Canine impaction and SABG timing

Among the 127 canines adjacent to the cleft assessed, 13 required surgical exposure, followed by orthodontic extrusion, while three severely impacted canines were extracted. All extractions occurred solely in the lateral agenesis group. The impaction risk was increased when SABG was performed at early mineralization stages (*R* ≤ 0,5). In contrast, 96,6% of canines erupted spontaneously into the bone graft when SABG was performed late (*R* ≥ 0,75).

### Canine axis-angle and eruption pattern

## Preoperative inclination

The axis angle was assessed using preoperative orthopantomograms.

Before SABG, the canine adjacent to the cleft with an existing lateral incisor exhibited a more upright position compared to those with lateral incisor agenesis, showing a statistically significant difference (Student’s t = 2,082).

In cases where no SABG was performed, the canine tended to erupt in an upright direction along the posterior cleft margin.

Statistical comparisons between mineralization stage subgroups were significant for both patient cohorts: Student’s t = 2,231 for regions with existing lateral incisors and Student’s t = 3,272 for those with lateral incisor agenesis.

## Post-eruption inclination

**Fig. 5:**
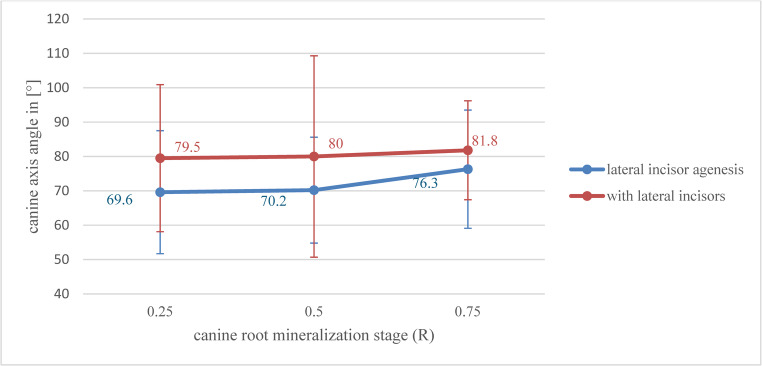
Arithmetic mean values (M) of canine axis-angles (in degrees º) after canine eruption according to R-values and SABG timing with 95% confidence intervals in the patient-cohort with lateral incisors / lateral incisor agenesis. (p < 0,05)

SABG timing did not influence the canine axis-angle in patients with lateral incisors. However, in agenesis cases, a moderate but statistically significant correlation was found between SABG timing and the axis-angle of the canine on the cleft side. When SABG was carried out during early mineralization stages (*R* ≤ 0,5), canines erupted in a more tilted position into the bone graft, favoring orthodontic space closure. At a later stage (*R* ≥ 0,75), the canine tended to erupt in a more upright position at the posterior cleft margin. The comparison between the two subgroups was statistically significant, with a Student’s t = 3,343. There was a mean canine inclination difference of 8,4 ° between the lateral incisor agenesis and non-agenesis patient cohorts. Overall, canines in non-agenesis cases exhibited higher axis angles and erupted more upright across all mineralization stages than in agenesis cases [Fig. 5].

After orthodontic treatment of patients with lateral incisor agenesis, the mean canine axis-angle was 91 ° following prosthodontic space closure and 81,3 ° following orthodontic space closure, resulting in a mean difference of 9,7 °, which was statistically significant (Student’s t = 5,702). In addition, surgical exposure and orthodontic traction of impacted canines did not negatively affect bone graft height. The results were even more favorable than those of spontaneously erupted canines. However, this difference was not statistically significant due to the small sample size of this cohort and should be interpreted with caution.

**Fig. 6:**
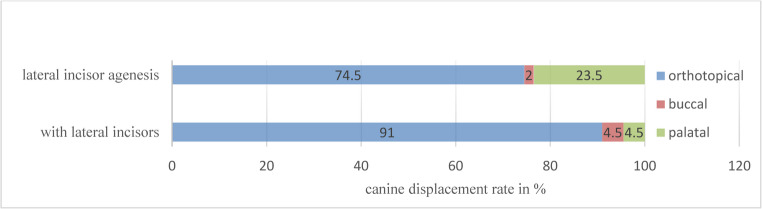
Canine eruption pattern (orthotopical, buccal/palatal displacement) in the transversal dimension according to the presence or agenesis of lateral incisors. (Significantly higher displacement rates observed for early SABG (*R* ≤ 0.5; *p* < 0,05))

Assessing the canine eruption pattern in a transverse dimension revealed that most (90,9%) canines erupt orthotopically in patients with lateral incisors. In agenesis cases, 24% were palatally, and 2% were buccally displaced. Hence, the timing of SABG plays an important role, as significantly higher displacement rates were observed when performed in early mineralization stages (*R* ≤ 0,5). A description of the transversal canine eruption pattern is shown in Fig. 6.

### Height of bone graft according to canine eruption and type of space closure

The height of the bone graft adjacent to the cleft, measured after the canine eruption, showed a statistically significant correlation with the timing of secondary osteoplasty in the lateral incisor agenesis group. When the R-value was *R*>0,5 at the time of the surgery, the bone graft height was significantly reduced, compared to cases with SABG at *R* ≤ 0,5 (Student’s t = 4,234). No such correlation was observed in the patient cohort with lateral incisors. A similar trend was noted when evaluating bone graft height in relation to the presence of lateral incisors. However, in lateral incisor agenesis cases, the type of space closure significantly influenced bone graft preservation. Patients treated with orthodontic space closure experienced significantly less bone resorption compared to those who underwent prosthodontic space closure (Student’s t = 5,406). Taking arithmetic mean values into account, higher rates of bone resorption after prosthodontic space closure (M = 5,8) were observed compared to orthodontic space closure (M = 3,3). However, the difference was not statistically significant. This discrepancy was likely due to natural bone atrophy resulting from the lack of functional loading in prosthodontically treated sites.

When orthodontic space closure was the treatment of choice, the bone graft height remained stable regardless of the agenesis of the lateral incisors, as presented in Tables 2 and 3. However, bone graft height was lower when SABG was performed late (*R* ≥ 0,75), particularly in lateral incisor agenesis cases, as evident from the mean values (e.g., *R* = 0,25: 1,8 vs. *R* = 1: 5,8).

**Table 2:** Bone graft height in patients with lateral incisors expressed, as a dimensionless ratio relative to canine crown length. M: arithmetic mean value; CI: 95%-confidence interval; n: number of cleft regions

type of space closure	after canine eruption			after orthodontic/prosthodontic treatment		
	**M**	**CI**	**n**	**M**	**CI**	**n**
prosthodontic	3,3	5,2	4	5,8	7,9	4
orthodontic	3,8	7,5	38	3,3	7,3	37
transposition	4,0	2,0	3	4,7	4,1	3
total	3,7	7,0	45	3,6	7,3	44

**Table 3:** Bone graft height in patients with lateral incisor agenesis expressed, as a dimensionless ratio relative to canine crown length. M: arithmetic mean value; CI: 95%-confidence interval; n: number of cleft regions

type of space closure	after canine eruption			after orthodontic/prosthodontic treatment		
	**M**	**CI**	**n**	**M**	**CI**	**n**
prosthodontic	4,3	3,9	40	6,0	3,6	36
orthodontic	2,5	5,0	39	2,5	5,1	25
total	3,4	4,7	79	5,3	5,6	61

### Height of the limbus alveolaris according to canine eruption and type of space closure

The height of the limbus alveolaris was measured mesial to the canine adjacent to the cleft.

Evaluating the height of the limbus alveolaris after canine eruption revealed a significantly (Student’s t = 4,221) lower bone level if SABG was performed late (*R* = 1) compared to surgical intervention in earlier root mineralization stages (*R* ≤ 0,75). In the earlier stages, limbus height differed only marginally and was independent of lateral incisor presence.

The type of space closure had a strong influence on limbus alveolaris height. Before orthodontic treatment, the bone level was well preserved in all groups, with bone resorption rates below 2 mm. This stability persisted in cases treated with orthodontic space closure, where limbus alveolaris height remained stable during and after treatment. No statistical association was found between limbus height and SABG timing. However, prosthodontic space closure was linked to significantly higher bone resorption in both groups:

Patients with lateral incisors (Student’s t = 4,434) and those with lateral incisor agenesis (Student’s t = 3,762).

### Periodontal evaluation

As part of a clinical follow-up, probing depths were measured mesial to the canines and distal to the central incisors adjacent to the cleft. The clinical examination revealed a healthy periodontal situation with probing depths < 3 mm. Neither lateral incisor agenesis nor the type of space closure had a statistically significant impact on periodontal health. However, slightly higher probing scores were assessed in cases with orthodontic space closure. Here, it is worth noting that the formation of an interdental papilla was not examined. No significant differences in probing scores were observed between the cleft and non-cleft sides in patients with UCLP.

### Vitality of cleft-sided canines

In this study, carbon dioxide snow (dry ice) was used for the pulp vitality testing. Only three canines showed no response to the test. One canine was endodontically treated and was in transposition with the lateral incisor. The other two non-responsive canines were part of a bridge construction used for prosthodontic space closure, resulting in unclear reactions during the vitality test.

## Discussion

The optimal timing of SABG remains a critical factor in graft integration, tooth eruption, and long-term orthodontic outcomes, yet it remains debated.

This study demonstrated that early SABG (*R* ≤ 0.5) favored orthodontic space closure and bone preservation, but increased the risk of canine impaction.

To our knowledge, this is the first study to systematically evaluate SABG timing using R-values, providing a developmental framework for individualized treatment planning.

As a single-center study, the outcomes primarily reflect the treatment protocol employed at Charité-Universitätsmedizin Berlin (Table 1). However, the survey by Khdairi et al. showed that the majority of European cleft centers follow a similar protocol, including maxillary expansion at the age of 9–10 years, followed by SABG using autologous bone harvested from the iliac crest and performed before canine eruption [22]. This approach supports the generalizability of the present findings. Regardless of the protocol adopted in cleft care, the surgeon’s experience remains a critical determinant of treatment outcomes, particularly for graft height and post-surgical complications, such as bleeding or dehiscence [23].

While stratification by cleft type and sex was considered, it was not performed in order to preserve statistical power. Instead, the patients were grouped based on the R-value, which is a more accurate marker that reflects dental developmental age more accurately than the chronological age, in patients with CLP, who often exhibit delayed and variable dental development [24]. The R-value can be assessed during routine preoperative radiological diagnostics without additional radiation exposure. Since canine eruption is closely linked to the root’s mineralization stage, it serves as a reliable developmental reference [25].

Additionally, the canine germ is located distal to the cleft and, unlike incisors, does not exhibit associated morphological aberrations, making it a reliable reference for root mineralization assessment [26]. The bone graft height and limbus alveolaris were evaluated relative to the canine crown. OPTGs obtained at T0, T1, and T2 were assessed following the method described by Aurouze et al. [27] and Long et al. [19]. The canine angulation in the mesiodistal dimension was assessed using the method outlined by Dausch-Neumann [20]. Modern X-ray devices equipped with positioning aids and staffs trained in their use ensure standardization and reproducibility of imaging. Positioning errors during OPTG acquisition were minimized by strict inclusion criteria and exclusion of low-quality images [18]. Despite the limitations of 2D imaging, previous studies have shown sufficient accuracy for assessing SABG outcomes [28, 29]. A continuous bone bridge of about 9 mm is considered essential for successful tooth eruption into the graft and effective orthodontic movement. Thus, precise radiologic examination around the erupting canine, in relation to individual dental development, is crucial for optimizing SABG timing [30, 31].

However, over the last years, CT and CBCT have become increasingly crucial for the assessment of cleft regions and bone grafts, enabling precise volumetric analysis, improving treatment planning and postoperative outcome assessment [32, 33]. Conventional 2D OPTGs often underestimate bone resorption [34] as they only capture the vertical dimensions. In this study, bucco-palatal resorption could not be accurately assessed from OPTGs. Resorption may reach up to 49,5% within the first year after surgery [35, 36], highlighting the importance of evaluating the bucco-palatal bone thickness to ensure proper canine positioning. Our study relies on conventional radiographic measurements while acknowledging the limitations of volumetric assessment compared to CBCT.

Although 3D imaging offers superior assessment compared to 2D, a consistent imaging protocol is necessary to ensure comparability across centers [37].

Furthermore, adherence to the ALARA (As Low As Reasonably Achievable) principle remains crucial, particularly in pediatric patients. Although studies acknowledge the surgical advantages of preoperative CBCT scans [38], recent research shows no clear predictive benefits of CBCT over conventional intraoral radiographs for assessing canine eruption following SABG [39]. Thus, advanced imaging should only be considered when its clinical advantages outweigh the risk of radiation exposure.

SABG, introduced by Boyne and Sands in 1972 [40], is typically performed during the late mixed dentition (8–12 years of age) and has become the standard treatment for reconstructing bony defects in CLP patients [41]. After age eight, maxillary growth is primarily completed, reducing the risk of growth impairment following SABG [42–44]. Bergland et al. [45] demonstrated optimal outcomes when SABG was performed before the canine eruption. In our cohort, only three out of 127 cleft areas were grafted before the age of eight, making growth effects unlikely to occur. Most patients (57%) underwent SABG between the ages of 9 and 11 (mean age: 9 ± 1,6 years), consistent with the previous reports (8,2 to 10,6 years) [35, 40–43].

### Impact of SABG timing on space closure

SABG timing significantly influenced space closure outcomes in patients with lateral incisor agenesis.

Early SABG (*R* ≤ 0,5) tripled the success rate of orthodontic space closures compared to late SABG (*R* ≥ 0,75), as the mesial drift of the erupting canine (*R* ≤ 0,5) decreased the canine inclination angle, and minimized orthodontic movement (Student’s t = 2,931). The required mesial movement for space closure ranged from only 1,7 mm (*R* = 0,25) to 6,2 mm (*R* = 1,0). Thus, early SABG (*R* ≤ 0,5) facilitates successful orthodontic space closure in CLP patients with lateral incisor agenesis. The absence or presence of lateral incisors and the mesio-distal space must be considered in decision-making on the type of space closure [46]. Although the aesthetic outcomes of both orthodontic and prosthodontic space closure are satisfactory and comparable in the long term, the risk of cranio-mandibular dysfunction increases after prosthodontic rehabilitation with resin-bonded bridges [45]. Despite conventional SABG being well documented and considered the gold standard, the current literature suggests a shift towards an earlier intervention before the eruption of the lateral incisors [47, 48].

Early SABG (around 5–7 years) may offer particular advantages for patients with CLP with lateral incisors, as it provides adequate bone support for their eruption [44]. Evidence, although limited, suggests that early SABG does not impair maxillary growth [9, 45, 49] and may yield superior outcomes, including significantly higher lateral incisor survival, improved bone graft volume [49] and better periodontal health [47]. Cephalometric and volumetric studies have shown that both groups exhibited similar maxillary growth patterns [50] between early and conventional SABG (around the age of ten), while some report greater bone preservation when grafting is performed earlier [40, 50]. Precious et al. [51] showed better periodontal health, crown length, and central incisor symmetry with early SABG. Importantly, regardless of the age at which the ABG is placed, the eruption of a permanent tooth (lateral incisor or canine) into the graft has a positive and stabilizing effect on bone height [40].

### Bone graft and limbus alveolaris height

This study highlights the impact of SABG timing on bone graft preservation in patients with lateral incisor agenesis. Late SABG (*R* = 1) showed 3,5 mm more resorption compared to early SABG (*R* = 0,25). These findings align with those of Bergland et al. [7], who reported that 64% of early SABG cases had preserved interdental septum height, compared to only 37% of late SABG cases (those performed before and after canine eruption, respectively). As septum height is crucial for future dental implants, early SABG may be preferable when orthodontic space closure is not indicated.

These findings align with previous studies [7, 52] that recommend SABG near the eruption of the tooth adjacent to the cleft, followed by early loading through orthodontic loading or implant placement, to prevent functional bone atrophy. Zhang et al. reported maximum bone graft reduction within three to six months post-SABG [43]. However, as CBCT was not yet standard, our assessments relied mainly on conventional 2D radiographs. Recent 3D studies emphasized the prognostic value of preoperative cleft volume in determining grafting success. Larger cleft defects have been consistently associated with increased risk of graft failure, stressing the need for precise volumetric assessment to guide treatment planning [53]. This has direct implications for graft material selection. Structural bone blocks, harvested from the iliac crest and chin, generally yield superior outcomes compared to cancellous bone [54]. In contrast, for smaller cleft defects, synthetic materials are gaining relevance. A recent evaluation of a bioactive glass material (GlassBONE™) demonstrated satisfactory bone fill, favorable periodontal health, and successful eruption patterns, supporting its potential as a minimally invasive alternative in selected pediatric patients [55].

### Canine impaction and eruption

Compared to the 1–2% prevalence of canine impaction in non-cleft populations [56], CLP patients are at a greater risk of cleft-sided canine impaction, with reports ranging between 6% and 56% [57–59]. In the present study, the overall impaction rate of cleft-sided canines was 10,2%, which falls within the abovementioned range. Notably, the majority (89,8%) of cleft-sided canines erupted spontaneously into the grafted alveolar region, revealing a positive effect of SABG on successful canine eruption, which aligns with the findings of a recent CBCT-based review [60]. Of the 127 cleft-sided canines evaluated, 13 were impacted, with three necessitating surgical removal. Notably, all of these cases occurred in patients with lateral incisor agenesis, underscoring the increased risk in this subgroup. Early SABG (*R* ≤ 0,5) was associated with an increasing impaction rate, consistent with the findings of Hoang et al. [58]. This trend was particularly evident in agenesis cases, which accounted for 76,9% of the impacted canines; more than three times the rate observed in patients with non-agenesis of lateral incisors.

Although early SABG (*R* < 0.5) was associated with increased impaction risk, this can be mitigated through close radiographic surveillance and timely interceptive orthodontic treatment. Early detection of eruption deviations is essential for guiding canine eruption.

Additionally, lateral incisor agenesis is associated with palatinal displacement of the erupting canine, likely due to the absence of the lateral incisor root, which guides the canine’s eruption path [61]. Consequently, lateral incisor agenesis has been identified as a potential risk factor for canine displacement and impaction after early SABG. Nevertheless, a systematic review by Lacerda et al. found only low evidence linking canine impaction to lateral incisor agenesis [62]. Additional predictors for canine impaction in cleft patients include advanced root development, apical tooth position, sharp axial angulation, older age at the time of SABG, and the need for regrafting [11, 63–65].

In our cohort, impacted canines were successfully managed with surgical exposure and orthodontic traction without adverse effects on graft stability or periodontal health. These results support a risk-benefit approach where early SABG can be performed safely with radiographic monitoring and interdisciplinary planning.

### Periodontal evaluation and interdisciplinary care

Periodontal stability and inflammation-free tissues are crucial for long-term graft success. Previous literature has shown that SABG does not adversely affect the periodontal integrity of teeth adjacent to the cleft, with attachment levels comparable to those of non-cleft teeth [66, 67]. In our cohort, probing depths of all cleft-sided teeth remained within the healthy range (< 3 mm) and did not differ significantly from those of the contralateral side in patients with UCLP.

Soft tissue outcomes such as papilla presence or gingival inflammation were not systematically recorded and therefore could not be included. Prospective studies are needed to evaluate these additional aspects of periodontal health. Loss of vitality was seldom observed and was not associated with either the surgical intervention or subsequent orthodontic treatment. Neither periodontal health nor vitality was correlated with the timing of SABG, the presence of lateral incisors, or the type of space closure. Poor oral hygiene is a known risk factor for surgical failure due to bone loss [68]. A 20-year follow-up study by Jabbari et al. demonstrated a positive correlation between the gingival bleeding index and prosthetic restorations in cleft areas, leading to increased bone resorption [69]. Given the increased susceptibility of cleft patients to periodontitis, thorough oral hygiene instructions, regular professional dental care, and strong interdisciplinary collaboration with restorative dentists and periodontists are crucial to ensure the long-term health and stability of the cleft region and minimize the need for regrafting.

### Limitations

Although this study provides important insight, some limitations should be acknowledged. The retrospective single-center study design may introduce selection bias and limit generalizability.

Although strict inclusion criteria and a uniform treatment protocol were applied, the retrospective design inherently limited randomization and prospective control. Furthermore, the radiographic assessment relied exclusively on 2D-imaging procedures, which cannot capture bucco-palatal dimensions or volumetric changes at the grafted site. Although OPTGs were the clinical standard during the treatment period and offered longitudinal comparability, they may under- or overestimate graft volume due to anatomical superimpositions. The absence of CBCT prevented evaluation of 3D graft morphology and spatial bone preservation. Future studies should incorporate CBCT-based volumetric analysis to validate and expand upon these findings, particularly by assessing long-term graft resorption and integration. Recent studies have already demonstrated the potential of CBCT-based volumetric methods to provide standardized assessments of graft outcomes, underscoring the importance of integrating such approaches in future research [70, 71].

Although the imbalance between agenesis and non-agenesis cases may limit the power of subgroup comparison, stratified analysis and careful subgroup evaluation were employed to minimize bias in interpretation. One limitation of this study is the inability to control for potential confounding variables such as cleft type (UCLP vs. BCLP), sex, and the presence or absence of lateral incisor agenesis using multivariate or regression analyses. Intra- and inter-examiner reliability of radiographic measurements (axis-angle, limbus height) was not formally assessed, which may limit reproducibility of the findings. Due to the retrospective nature of the dataset and inconsistencies in clinical documentation over time, applying such statistical models without introducing bias was not feasible. Nevertheless, these variables may influence graft outcomes and should be taken into account when interpreting the results. Future prospective studies with standardized protocols and larger sample sizes are essential to multivariable statistical analyses and to clarify the influence of these confounding variables on treatment outcomes.

## Clinical implications

Treatment decisions should balance the benefits of early SABG regarding graft stability and orthodontic space closure with potential tooth eruption risks. The findings underscore the need for individualized and interdisciplinary treatment planning based on the developmental stage (R-value), cleft morphology, and agenesis status.

For patients with lateral incisor agenesis, early SABG is recommended when orthodontic space closure is planned, provided that careful monitoring for potential canine impaction is ensured.

In contrast, when prosthodontic rehabilitation is anticipated, later SABG may better preserve bone volume.

To strengthen the clinical evidence base, future trials should adopt a prospective, multicenter design with larger, demographically balanced cohorts. Incorporating 3D imaging modalities such as CBCT and standardized protocols for radiographic and periodontal examinations would enhance comparability across centers. Furthermore, stratification by cleft type and tooth agenesis status, combined with multivariate statistical models, may further clarify the complex interaction between SABG timing, dental development, and long-term outcomes.

In clinical practice, dental developmental age (R-value) should therefore be prioritized over chronological age when determining the optimal timing for SABG.

## Conclusions

SABG timing is a decisive factor in treatment outcomes in patients with CLP. Using R-value as a developmental reference provides a reliable method for optimizing graft timing. Performing SABG during early canine mineralization stages (*R* ≤ 0,5) promotes bone preservation and facilitates orthodontic space closure, especially in patients with lateral incisor agenesis. Furthermore, bone resorption in the graft and adjacent to the limbus alveolaris was significantly lower following orthodontic space closure compared to prosthodontic treatment alternatives.

Early SABG, at the *R* ≤ 0,5 stage, may slightly increase the risk of canine impaction or transposition with the lateral incisor. However, these complications can be effectively managed through surgical canine exposure and orthodontic extrusion without leading to higher bone resorption rates or periodontal damage.

While our findings reflect a single-center experience, the alignment of our treatment protocol with those used across European cleft centers supports their broader applicability.

In conclusion, this study underscores the crucial role of precisely tailored and optimally timed SABG in optimizing functional and structural outcomes in cleft site management while minimizing potential complications.

Future research should integrate CBCT-based volumetric assessment with additional clinical and biomechanical parameters in order to refine treatment protocols and enhance outcomes for patients with CLP.

##  Appendix A: checklist of the STROBE guidelines[72]

**Figure:**
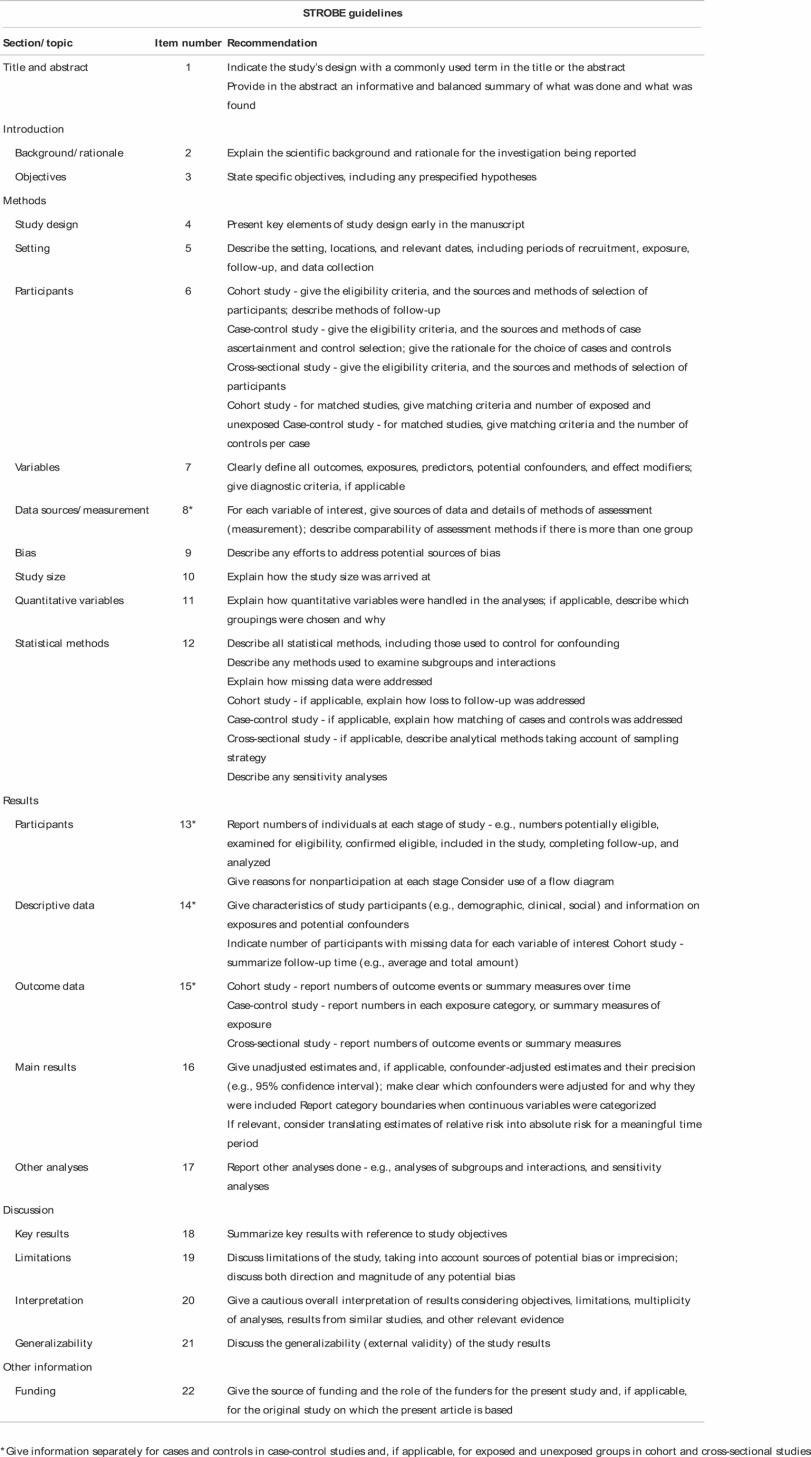

## Data Availability

No datasets were generated or analysed during the current study.
